# A novel geo-hierarchical population mobility model for spatial spreading of resurgent epidemics

**DOI:** 10.1038/s41598-021-93810-8

**Published:** 2021-07-12

**Authors:** Alexandru Topîrceanu, Radu-Emil Precup

**Affiliations:** 1 Department of Computer and Information Technology, Politehnica University Timisoara, Timisoara, Romania; 2 Department of Automation and Applied Informatics, Politehnica University Timisoara, Timisoara, Romania

**Keywords:** Computational science, Computational models

## Abstract

Computational models for large, resurgent epidemics are recognized as a crucial tool for predicting the spread of infectious diseases. It is widely agreed, that such models can be augmented with realistic multiscale population models and by incorporating human mobility patterns. Nevertheless, a large proportion of recent studies, aimed at better understanding global epidemics, like influenza, measles, H1N1, SARS, and COVID-19, underestimate the role of heterogeneous mixing in populations, characterized by strong social structures and geography. Motivated by the reduced tractability of studies employing homogeneous mixing, which make conclusions hard to deduce, we propose a new, very fine-grained model incorporating the spatial distribution of population into geographical settlements, with a hierarchical organization down to the level of households (inside which we assume homogeneous mixing). In addition, population is organized heterogeneously outside households, and we model the movement of individuals using travel distance and frequency parameters for inter- and intra-settlement movement. Discrete event simulation, employing an adapted SIR model with relapse, reproduces important qualitative characteristics of real epidemics, like high variation in size and temporal heterogeneity (e.g., waves), that are challenging to reproduce and to quantify with existing measures. Our results pinpoint an important aspect, that epidemic size is more sensitive to the increase in distance of travel, rather that the frequency of travel. Finally, we discuss implications for the control of epidemics by integrating human mobility restrictions, as well as progressive vaccination of individuals.

## Introduction

Understanding the dynamics of large, resurgent epidemics is an ongoing scientific effort aimed at controlling and preventing the spread of infectious diseases. Disease epidemiology, computational epidemics, and network science are some of the major scientific fields involved in this high impact social challenge. Notable research has been conducted over the past 30 years, answering important questions on the processes driving epidemics, and proposing strategies for prediction and control^1–4^. The heavy socio-economical burden of epidemics has been demonstrated repeatedly during crises like SARS^5^, Ebola^6^ or recent COVID-19^7^. To this end, we need to be able to predict long-term epidemic evolution, and the impact of governmental interventions, like isolation, travel restrictions, and vaccination/immunization of the population^8–12^.

In light of these challenges, we find recent studies that are predominantly augmenting mass-action models into tools suitable for analyzing large scale epidemics^8,12–16^. However, in most cases, we notice that their underlying epidemic models (e.g., SI, SIS, SIR, SEIR, SIRS) adopt homogeneous mixing of the population (i.e., all individuals are fully connected inside single scale compartments or stochastic blocks)^15–19^. Also underpinned by homogeneous mixing models, we find many *flattening the curve*-type solutions that try to reduce the reproduction number $R_0$; on the other hand, $R_0$ is found to have little influence on the final size of large-scale epidemics^20^, as well as being hard to estimate in a real-world context^21^. The over-simplification of social organization lacks the complexity of global scale population organization^22^, which is dictated by geographical, historical, demographic and economic factors. Consequently, numerical simulation of such simplified models can lead to over- or under-estimations in terms of epidemic size^10,16,23^ or duration^8,12,15,17^.

Conversely, we find some important studies which developed more robust and realistic models for epidemic dynamics and contagion, for heterogeneous population organization and human mobility. Without a doubt, the structure of networks is found to be paramount in explaining infectious spreading patterns^4,22^ seen in empirical data for transmissible diseases such as SARS^24^, influenza^16,21^, measles^25^, or HIV^1^. Community structure is a known key factor influencing the speed of epidemics. Chen et al.^26^ show that overlapping in communities leads to increased infection prevalence and a higher spread velocity in the early stages of emerging infections; Salathé et al.^4^ show that the dynamics of epidemics is influenced by the structure of communities, which, in turn, has implications on immunization strategies for large epidemics; Shang et al.^27^ show that overlapping communities and a higher network average degree accelerate spreading; Stegehuis et al.^28^ show that the structure of communities has a significant influence on the behavior of percolation on networks, as community structure can stimulate or suppress spreading, based on the mesoscopic set of communities.

Further incorporating human mobility and contact patterns increases the realism of an epidemic model. We note the work of Liu et al.^21^ which shows that the reproduction number $R_0$ has a much higher variability than expected, due to the heterogeneity of contact networks. Fueled by a data driven approach, the authors propose a multiplex representation of the population. Mistry et al.^22^ provide accurate age-stratified contact matrices for a large number of countries further motivating the need for a heterogeneous approach in disease modeling. Sattenspiel et al.^29^ extend a SIR model with five fixed patterns of mobility, but use otherwise large compartments for modeling the population structure. Watts et al.^20^ propose a fully synthetic hierarchical block model, aimed at reproducing multiple epidemic waves, but without integrating realistic distances between communities, or any correlation to real-world human settlement organization. Additionally, Salathé et al.^30^ analyze contact networks in the USA and confirm that heterogeneity is essential on the larger scale, but it is reliable enough to assume homogeneous contact inside small communities (like high schools). This conclusion reinforces our choice for partitioning the population into very fine-grained communities (to the level of households), and further simplification of the small communities’ topology, from a social network, to a stochastic block model^31^.

We also note the recent work of Calvetti et al.^32^ which adapt a network SEIR model using a single scale lattice of geographical blocks for modeling spatial mobility of the population. By contrast, our approach models a multiscale population with much higher granularity. Finally we note our previous work^33^, which is a first attempt at exploring geo-spatially organized populations. However^33^, is limited to studying the impact of country density on epidemic spreading on a single scale population model.

The motivation of this study is to describe a robust epidemic modeling framework which simultaneously incorporates accurate population modeling and human mobility, both of which represent ongoing challenges due to their theoretical complexity as well as limitation in available data for validation. Thus, we propose the novel geo-hierarchical population mobility model (GHPM) which lies at the crossroads of population organization and mobility, both of which are key aspects to consider when targeting realistic large-scale resurgent epidemic outbreaks. We propose the novel idea of distributing a population into spatially organized communities (i.e., human settlements), which are then organized into a hierarchy of administrative divisions (i.e., district, neighborhood, street, block, household). Thus, the population is partitioned with very high granularity all the way down to household-/family-level, containing just a few individuals, but where the transmission risks are highest^34^. Embedded into our population model, we further propose a novel mobility algorithm based on the geographical distance between settlements and their size, which determines the complexity of the underlying hierarchical structure. Finally, targeting the reliable reproduction of resurgent epidemics, we analyze the complex interplay between the population mobility model of GHPM and outbreak dynamics by adopting a modified SIR model with patient relapse. Altogether, the resulting framework is analyzed using detailed computer simulations.

In contrast to other computational models like GLEaMviz^35^, RAPIDD Ebola forecasting^36^, or^37^, our GHPM model is, to the best of our knowledge, the first framework to combine a geo-spatial and a hierarchical model to structure population, alongside an epidemic model with relapse. Using available empirical data on influenza and COVID-19, we show how GHPM reproduces similar epidemic dynamics (e.g., size, waves). The main focus of this paper is to determine how the population organization, travel distance and travel frequency affect the spread of diseases on large scales (country-level), and how restriction and immunization strategies can be applied efficiently to control epidemics.

## Results

### Characteristics of large epidemics

Real-world large, resurgent epidemics are known to be shaped by repeated waves of non-deterministic amplitude, and an overall limited epidemic size $\phi$ (it is safe to assume $\phi <0.1$ considering epidemics over the last decades^38,39^). To this end, we investigate the epidemic sizes of various outbreaks over heterogeneous geographical areas. Since many infectious diseases are repetitive, seasonal (e.g., influenza, pertussis), and others appear and are then permanently eradicated (e.g., H1N1, smallpox), our approach, throughout the rest of the study, is to use the yearly epidemic size $\phi _y$ as a measure of the repeating outbreak waves. Therefore, we further provide a statistical overview of seasonal, eradicated and ongoing epidemics worldwide, as such: seasonal influenza in Germany (2014), influenza in California (winter season 2016/2017), global H1N1 cases (2009), measles in Indonesia (2016), Pertussis in California (2010), and data on COVID-19, with estimates on global epidemic sizes for 2020.

**Figure 1:**
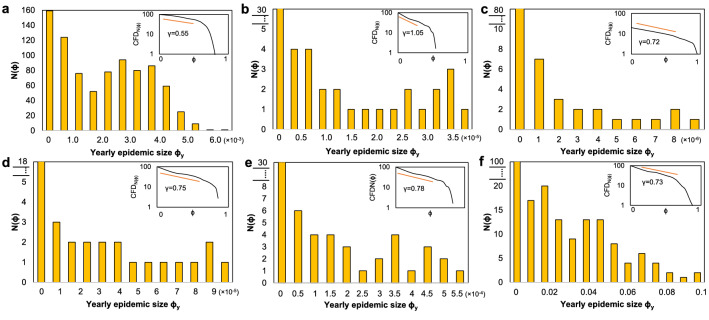
Yearly epidemic size $\phi _y$ distributions normalized by the population of each administrative region for: influenza in counties of Germany (**a**), influenza in counties of California (**b**), global H1N1 (**c**), measles in Indonesia (**d**), pertussis in California (**e**) and global COVID-19 cases (**f**). Representative modes are detected, in all examples, for small outbreaks (e.g., $\phi _y<0.02$ in f) followed by a relatively uniform distribution of larger epidemic sizes (e.g., $0.02< \phi _y <0.06$ in f). The insets summarize the cumulative frequency distributions CFD, quantifying the amount of epidemics sized $\le \phi _y$, on log-log axes. Fitting a power-law over the flatter regions (orange line) results in exponents $\gamma =0.55-1.05$.

The distributions of the yearly epidemic sizes $\phi _y$ are given in Fig. 1 [COVID-19 in panel (f)], showing predominantly small-sized epidemics [$N(\phi _y)$ is highest for smaller outbreak sizes, like $\phi _y < 0.02$ in panel (f), for COVID-19]. The power-law fits for the flatter area in each distribution [e.g., $0.02< \phi <0.06$ in panel (f)] range within $\gamma =0.55-1.05$, well outside the representative power-law exponent $2< \gamma <3$; this means that larger sized epidemics are not exceptional situations. Nevertheless, the upper yearly real-world threshold for epidemic sizes seems to be small^39^, e.g., $\phi < 4 \times 10^{-4}$ for influenza in California, or $\phi < 0.1$ for COVID-19. Even though COVID-19 is an ongoing pandemic, with final size and dynamics not yet known, the statistical analysis shows that the regional epidemic size distribution is consistent with historical data on other outbreaks.

We observe that, in case of large and/or long-lasting epidemics, we find heterogeneity and multi-modality in outbreak size over a heterogeneous geographical area (i.e, world’s countries, regions in Indonesia, counties in California, Germany). Since regional diversity (e.g., demographics, climate, mobility, NPIs) is highly relevant in determining seasonality and resurgence of epidemics^40^ we are encouraged to further develop a multiscale population structure, based on real-world geographic data.

### The geo-hierarchical population mobility model (GHPM)

#### Population structuring

Despite of the political, historical and geographical factors influencing the boundaries of countries and states, human populations have always flocked together into settlements^41^. As such, our proposed GHPM model starts from a targeted, real-world geographical area *A* (e.g., a continent, a country, a state), quantified by a set *S* of (real) settlements, which represent the basis of human organization and cooperation. In contrast to the abstract representation of network communities, each settlement $s_i \in S$ is modeled according to real-world data, using its geographical position $s_i(x_i, y_i)$ (i.e., longitude, latitude) and estimated size $\Omega ^*(s_i)$ (i.e., number of individuals).

Next, we create a multi-level hierarchical structure under each settlement based on its estimated population $\Omega ^*(s_i)$. This hierarchy of administrative divisions is summarized in Table 1, with the lowest level corresponding to households, each containing a family of average size $h_{size}$^42^ (more details in “Methods”). The number of households $n_h$ for a settlement is estimated as $n_h=\Omega ^*(s_i)/h_{size}$, where $h_{size}$ is given by a distribution (see SI.1). From *households* upward, in the administrative hierarchy of each settlement, we define *blocks*, whose number $n_b$ is given by the number of households as $n_b=(n_h)^\beta$, followed by *streets* ($n_s=(n_b)^\beta$), *neighborhoods* ($n_n=(n_s)^\beta$), *districts* ($n_d=(n_n)^\beta$), and finally the root of the hierarchy represented by the settlement. The impact of choosing different branching factors $\beta$ is detailed in SI.2. Also, note that the number of hierarchical levels added to each settlement depends on its estimated size $\Omega ^*(s_i)$ as defined in Table 1. Thus, we differentiate between the complexity of population organization in villages, towns, cities and metropolises (with 2–5 levels accordingly).

**Table 1:** Summary of the possible hierarchical administrative divisions characterizing a settlement $s_i$, given its estimated real-world population $\Omega ^*(s_i)$. Each division models a distinct hierarchical level in the settlement; based on its size, a settlement is modeled with 2–5 hierarchical levels (village–metropolis).

Administrative division	Applies to	Population	Inclusion criteria	Amount/$s_i$	Level
Settlement	–	–	yes, given size $\Omega ^*(s_i)$	1	6
District	Metropolis	Highest	if $\Omega ^*(s_i)\ge 1M$	$n_d=(n_n)^\beta$	5
Neighborhood	$\ge$ city		if $\Omega ^*(s_i) \ge 100K$	$n_n=(n_s)^\beta$	4
Street	$\ge$ town	$\uparrow$	if $\Omega ^*(s_i) \ge 10K$	$n_s=(n_b)^\beta$	3
Block	$\ge$ village		any, $\Omega ^*(s_i)>0$	$n_b=(n_h)^\beta$	2
Household	All settlements	Lowest	any, $\Omega ^*(s_i)>0$	$n_h=\Omega ^*(s_i)/h_{size}$	1

**Figure 2:**
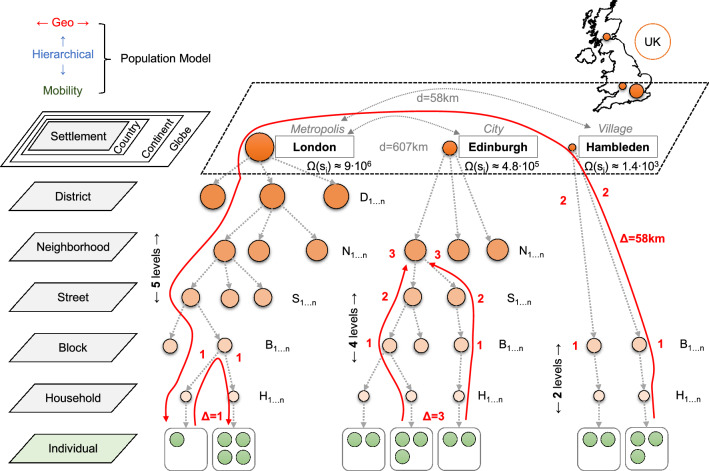
Conceptual representation of the hierarchical population structure of three settlements in the UK with real-world position and population. On the left side, all possible administrative divisions are enumerated; in the central panel, the hierarchical levels of each settlement are defined (orange nodes, based on the population $\Omega (s_i)$), down to the level of households which contain a small, random number (see “Methods”) of individuals (green nodes). The red arrows suggest three different mobility scenarios: (London) nodes move from one household to another with travel distance $\Delta =1$; (Edinburgh) nodes moves from a household to the neighborhood level with travel distance $\Delta =3$; (Hambleden) nodes move from one household to a random household in London, with travel distance $\Delta =58$ km.

Once the hierarchy of administrative divisions is created, each household $h_j$ in $s_i$ is randomly populated with individuals based on $h_{size}$. The final size of a household is represented as $\Omega (h_j)$ and the final population of each parent division is calculated as the sum of the population of the households in the parent’s subtree. As such, the final population $\Omega (s_i)$ (without the $^*$ symbol) of the settlement becomes $\Omega (s_i) = \sum \Omega (h_j)$; likewise, the total population of *A* becomes $\Omega =\sum \Omega (s_i)$. We also introduce the notations $D_i$, for the set of all divisions inside any settlement $s_i \in S$ (note that $|D_i|=n_h+n_b+n_s+n_n+n_d$), and $H_i$, for the set of all households in any $s_i \in S$ (note that $|H_i|=n_h$, $H_i \subset D_i$). This concludes the definition of the geo-hierarchical population structure, which is depicted in Fig. 2, using three settlements from the United Kingdom as an example. For more details on the experimental setup of GHPM refer to the “Methods” section.

#### Individual mobility

The mobility algorithm is described by a stochastic mobility function, based on the previously obtained geo-hierarchical population structure. The algorithm is applied to every individual $n_k$, from all settlements $s_i \in S$, for every iteration of the simulation time *t*. An individual can adopt one of three different movement scenarios:

1. Travel to another settlement $s_j \in S$ with probability $P_1$, in a randomly chosen household $h_j$ of that settlement. Furthermore, for a random timeout of 1–10 iterations (e.g., days), the individual is part of $h_j$, after which, the individual is returned to his original household. We use this simple timeout mechanism to implement the idea of business/leisure travel for a random, limited duration.
2. Otherwise, remain in the same settlement $s_i$ with probability $P_2$, and move to another division $div_j \in D_i$ (a household, or any upper level division inside the settlement). Furthermore, the individual is part of $div_j$ for 1 iteration (e.g., day), after which, the individual is returned to his original household. We use this simple timeout mechanism to implement the idea of short local trips to work, shopping etc.
3. Otherwise, remain home with probability $P_3$. The individual does not move out of his household.

The mechanisms of inter-settlement travel (scenario 1) and intra-settlement travel (scenario 2) are based on the same principle—a probability proportional to the population of the target settlement/division, and indirect proportional to the distance traveled. As such, the absolute inter-settlement travel probability $p_{inter}^*(s_i,s_j)$ is:

1 $$\begin{aligned} p_{inter}^*(s_i,s_j) = {\left\{ \begin{array}{ll} \Theta \cdot e^{\textstyle - \frac{\Delta (s_i,s_j)}{\Psi \; \cdot \; lg \Omega (s_j) } }, &{} \text {if}\, s_i \ne s_j, \; \forall i,j: 1\le i,j \le |S|\\ 1, &{} \text {if }\, s_i = s_j \end{array}\right. } \end{aligned}$$

where $\Delta$ is the geographical Euclidean distance between the two settlements (in km, based on latitude and longitude), *lg* is the log base 10 of the population of the settlement to travel to, $0 < \Psi \le 1$ is a tunable *travel distance* parameter, and $0< \Theta \le 1$ is a tunable *travel frequency* parameter. The effects of these two parameters are detailed in the forthcoming experimental results sections and the *Discussion*. The actual probability $p_{inter}(s_i,s_j)$ of travel between two settlements is obtained through normalization of $p_{inter}^*(s_i,s_j)$ as:

2 $$\begin{aligned} p_{inter}(s_i,s_j) = p_{inter}^*(s_i,s_j) / \sum _{\forall k=1}^{|S|} p_{inter}^*(s_i,s_k) \quad \mathrm {with}\quad \sum _{\forall k=1}^{|S|} p_{inter}(s_i,s_k)=1 \end{aligned}$$

Based on Eq. (2), if an individual leaves its home settlement $s_i$, he will be associated to a randomly chosen household $h_j$ in the target settlement $s_j$. If the individual does not leave the settlement (based on Eq. 2), we compute a similar intra-settlement probability $p_{intra}^*(h_i,div_j)$ of mobility between a household $h_i \in H_i$ and any other administrative division $div_j \in D_i$ (e.g., household, block, street etc.) from within $s_i$ as:

3 $$\begin{aligned} p_{intra}^*(h_i,div_j) = {\left\{ \begin{array}{ll} \Theta \cdot e^{\textstyle - \frac{\Delta (h_i,div_j)}{\Psi \; \cdot \; lg\Omega (div_j) } }, &{} \text {if}\, h_i \ne div_j, \; \forall i: 1\le i \le |H_i| \;\mathrm {and}\; \forall j: 1\le j \le |D_i|\\ 1, &{} \text {if }\, h_i = div_j \end{array}\right. } \end{aligned}$$

where, similar to Eq. (1), we use the distance $\Delta$, population $\Omega$, the travel frequency parameter $\Theta$, and the travel distance parameter $\Psi$. Inside a settlement, $\Delta$ is the maximum distance to the lowest common ancestor of the two divisions. See Fig. 2 with examples $\Delta =1$ (London) and $\Delta =3$ (Edinburgh). When $div_j$ is another household, then $\Omega (div_j)$ is equivalent with the size of the household $\Omega (h_j)$; for $div_j$ being an upper level division, $\Omega (div_j)$ is the sum of sizes of all households under that division. The actual intra-settlement probability $p_{intra}(h_i,div_j)$ of travel between a household and another division is obtained through normalization of $p_{intra}^*(h_i,div_j)$ as:

4 $$\begin{aligned} p_{intra}(h_i,div_j) = p_{intra}^*(h_i,div_j) / \sum _{\forall k=1}^{|D_i|} p_{intra}^*(h_i,div_k) \quad \mathrm {with}\quad \sum _{\forall k=1}^{|d_i|} p_{intra}(h_i,div_k)=1 \end{aligned}$$

Based on Eqs. (1)–(4), any individual will choose one of the three mobility scenarios with the following probabilities: $P_1 = p_{inter}(s_i,s_j)$ for scenario 1, $P_2 = (1-p_{inter}(s_i,s_j))\cdot p_{intra}(h_i,div_j)$ for scenario 2, and $P_3 = (1-p_{inter}(s_i,s_j))\cdot (1-p_{intra}(h_i,div_j))$ for scenario 3. Adding up the three probabilities $P_1+P_2+P_3 = 1$.

As a final observation, we consider that while an individual is “away” from home (scenarios 1, 2), he is exempt from any further travel until he returns to his original household. Also, the starting point of travel for any individual is its original household.

#### Epidemic transmission

We intend to use the GHPM model to replicate resurgent epidemics, like influenza or COVID-19 (see Fig. 1), so that we adopt a SIR epidemic model with relapse^43^, also know as a SIRI model^44^. Hence, we make the following assumptions:

- Each individual can be in one of three mutually exclusive states (susceptible *S*, infected *I*, or recovered *R*), where the fraction of the population in each state, at any discrete moment in time *t*, is denoted as *S*(*t*), *I*(*t*), and *R*(*t*), respectively. At every time step, an infected coming in contact with a susceptible individual, can transfer the disease with a probability $\lambda$. Subsequently, an infected individual can recover with a probability $\mu$, after which he remains recovered, but only for a specific period (see “GHPM experimental setup” for details on the reproduction number estimation). After this timeout period, the individual becomes susceptible again.
- Homogeneous population mixing is sufficiently accurate at the small granularity of households^30,34^, as well as for short duration (1 day) in higher administrative divisions (e.g., block, neighborhood). Moreover, we consider that epidemic contagion only occurs between infected and susceptible individuals found in the same administrative division at the same time *t*. Given $R_0>1$ as a necessary condition to trigger an epidemic, we choose $R_0=3$; nevertheless, this does not guarantee that an outbreak will occur every single simulation.
- Only one random individual (seed) is infected at $t=0$. Once triggered, an epidemic can develop inside the household of the seed using homogeneous mixing with the described SIR model. Further spatial spreading depends entirely on the described mobility algorithm.

### Estimating epidemic dynamics

We run the GHPM model in various scenarios, defined by different parameter settings, to better understand the potential of recent epidemic outbreaks to exhibit heterogeneous and resurgent behavior.

**Figure 3:**
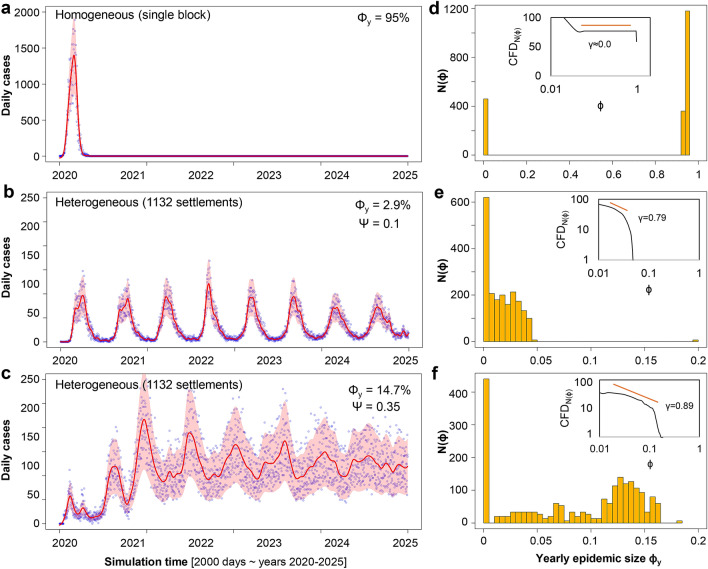
Aggregated results proving the resurgent behavior of epidemics in our simulation experiments. (**a**–**c**) Representative time series of new daily cases for (**a**) homogeneously mixing population, and (**b**,**c**), heterogeneously structured population using GHPM. In (**a**), most of the population is quickly infected ($\phi _y=95\%$), as a typical outbreak surges rapidly only once and then drops back to zero. By contrast, in (**b**,**c**), the two epidemics exhibits visible resurgence, and infect very different (and smaller) proportions of individuals ($\phi _y=2.9\%$, and $\phi _y=14.7\%$). (**d**–**f**) Corresponding epidemic size distributions for all three depicted scenarios in (**a**–**c**). We ran 2000 simulations each, on a population model of Germany with 533,160 individuals and simulation time $t=2000$ days. (**d**) Strictly bimodal epidemic size $\phi _y$ distribution on a homogeneously mixing population (all individuals placed in one single-scale settlement). (**e**,**f**) Population is structured according to GHPM, and the two setups differ in the travel distance parameter $\Psi$ (0.1 and 0.35). Both (**e**) and (**f**) depict distributions of similar size with modes near $\phi _y = 0$, and an approximately uniform distribution for $0<\phi _y<0.2$. From a qualitative standpoint, both distributions are comparable to real epidemic data in Fig. 1. The insets summarzie the cumulative frequency distributions CFD. Fitting a power-law over the flatter regions (orange line) results in exponents $\gamma = 0.79-0.89$ for the GHPM model.

In Fig. 3 we investigate the potential of our GHPM model’s heterogeneity in estimating epidemic dynamics. As such, we vary the travel distance parameter $\Psi$ (while keeping all other parameters fixed). Figure 3d depicts a bimodal distribution of the yearly epidemic size $\phi _y$, given simple homogeneous population mixing, modeled through one single-scale settlement ($\Omega =533,160$ individuals). Alternatively, Fig. 3e,f depict two epidemic size distributions achieved by GHPM with its specific hierarchical structure and mobility (same population $\Omega$, $R_0=3$, simulation time $t=2000$ iterations (days), $\Psi =0.1$, respectively $\Psi =0.35$). Regardless of the epidemic sizes, a visible mode is detected near $\phi _y=0$, followed by a relatively flat distribution. Figure 3b,c shows two representative simulated epidemics generated with GHPM for the corresponding travel distance parameters $\Psi =0.1$, and $\Psi =0.35$. Figure 3a depicts the same time series information corresponding to the homogeneously mixing model in Fig. 3d.

Two main observations can be summarized from Fig. 3. First, the homogeneous model in panel (a) reproduces an over-simplified spike in the number of daily cases without any resurgence, while panels (b-c) display different shapes and sizes $\phi _y$, as well as resurgence. Even if the epidemic looks like it repeatedly dies out in panel (b), it manages to flare up again in new waves. Second, the homogeneous model in panel (a) is only capable of reproducing a bimodal epidemic size distribution. Nevertheless, real data (see Fig. 1) confirms the variability and resurgence reproduced by our GHPM heterogeneous model.

These results also have implications in the context of epidemic control. Even though the two scenarios depicted in Fig. 3b,c (with corresponding distributions in Fig. 3e,f) have a similar parameter setting, the difference in travel distance $\Psi$ results in a large variation of epidemic size. As $\Psi$ increases, the expected epidemic size $\phi$ increases and the distribution tends to become more bimodal. Conversely, for very small $\Psi$, the epidemic size distribution tends to have one single mode near 0. Thus, extremes of the travel distance parameter $\Psi$ lead to entirely local or entirely global outbreaks, and GHPM can be compared with a homogeneous one. However, when $\Psi$ is neither too small or too large (like in Fig. 3b,c), we obtain the most faithful reproduction of real epidemic dynamics (Fig. 1c,d), and the impact of a fixed $R_0$ is negligible.

### The impact of travel distance versus travel frequency

**Figure 4:**
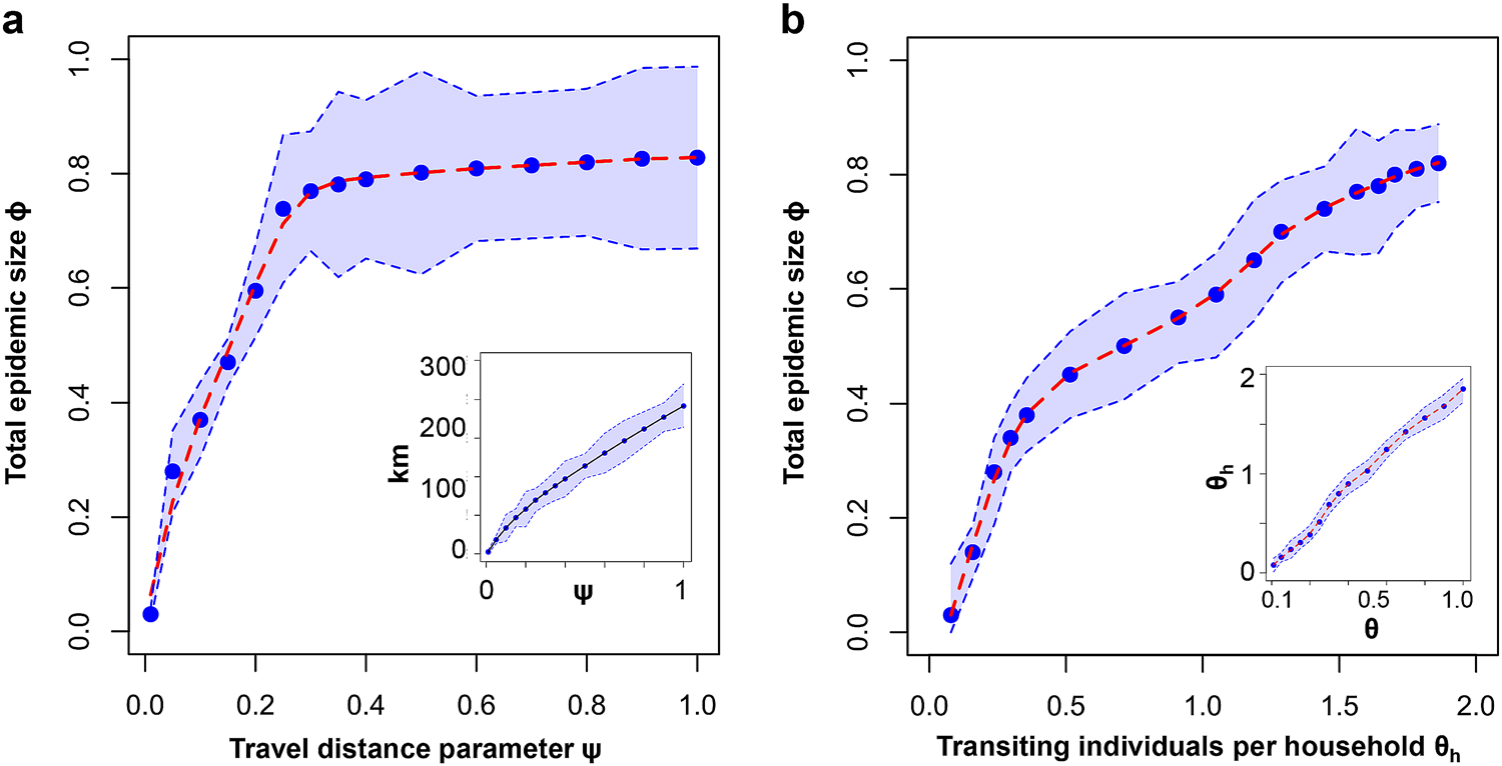
Total epidemic size $\phi$ averaged over 2000 simulations, (**a**) as a function of the travel distance parameter $\Psi$, respectively (**b**) as a function of the travel frequency, normalized at household level $\Theta _h$. (**a**) Epidemic size $\phi$ exhibits a clear phase transition starting with minimal increases in $\Psi$, which, in turn, enables non-local spreading. (Inset) Linear relationship between $\Psi$ and the daily travel distance (in km), averaged over all individuals in the GHPM model for Germany (sized roughly 600 $\times$ 750 km). (**b**) Epidemic growth shows no visible phase transition in response to increases in $\Theta _h$. (Inset) Linear relationship between $\Theta$ and $\Theta _h$, averaged over all households. The blue regions indicate 95% CI.

Figure 4a summarizes the impact of increasing the travel distance parameter $\Psi$ on the total epidemic size $\phi$. In accordance to Eqs. (1) and (3), $\Psi$ influences the range over which individuals are likely to travel, both outside and inside settlements. Namely, a higher $\Psi$ increases the probability of any individual to travel to a more distant settlement (or division) from its original household, because it directly reduces the weight of $\Delta$ in Eqs. (1) and (3).

Figure 4b depicts $\phi$ as a function of the travel frequency parameter $\Theta$. In addition, we choose to introduce $\Theta _h$ as the average number of individuals transiting (leaving) a household, which is a more intuitive measure than $\Theta$ alone. Unlike $\Theta$, which is an input parameter in the model, the value of $\Theta _h$ is measured during simulation, and normalized by the number of households. The inset of Fig. 4b shows the linear relationship ($\Theta$, $\Theta _n$). In accordance to Eqs. (1) and (3), $\Theta$ affects the weight of each probability of any individual to travel outside its home settlement (or household).

In case of Figure 4a, we observe a clear phase transition from local to global epidemic as $\Psi$ increases. Intuitively, when the two travel parameters are close to 0, infected individuals are more likely to remain in their original household (or settlement); as such, the resulting epidemic size $\phi$ is delimited to the local scale of $h_{size}$. As long as the size of a modeled local scale is much smaller than the size of the entire population, truly large epidemics cannot occur with $\Psi ,\Theta ,\Theta _h \rightarrow 0$. On the other hand, When $\Psi > 0.01$ and $\Theta _h>0.01$, large scale outbreaks can occur, regardless of the modeled population size.

When either $\Psi$ or $\Theta _h$ converge towards extremes (0 and 1) GHPM can be assimilated to a homogeneously population model, where the set of susceptible individuals coincides with the size of a household $h_{size}$ and $\Omega$, correspondingly (stochastic dynamics appear at the household level, or at the global scale). Nevertheless, neither of these limits (0 and 1) are realistic representations of real-world mobility, which is a mixture of local and non-local patterns. As such, we further focus on the intermediate values of travel parameters where shifts in $\phi$ are visible. The phase transition in Fig. 4a is best fitted by a 3rd degree polynomial, whereas the less abrupt increase in $\phi$ triggered by $\Theta _h$ is best modeled by a linear fit. This suggests that small changes in $\Psi$ have a much greater impact on the size of the epidemic $\phi$ compared to similar changes in $\Theta _h$. Similarly, by considering the linear relationship between ($\Theta$, $\Theta _h$), depicted in the inset of Fig. 4b, we conclude that the epidemic size $\phi$ is more sensitive to increases in $\Psi$ than increases in $\Theta$.

The analysis of travel parameters in the GHPM model leads to an insightful conclusion with possible effective policy implications on the ongoing COVID-19 pandemic and future outbreaks. We found that restricting the distance, rather than the frequency of travel – during an outbreak—is the more adequate approach to minimize the eventual impact of an epidemic.

### Embedding mobility restrictions and immunization

Finally, we compare the effectiveness of mobility restrictions versus mass immunization. The first, is implemented by long distance travel restrictions, a measure already adopted world-wide, throughout 2020, during the COVID-19 pandemic, and with notable results^45,46^. In GHPM, this restriction is obtained by reducing the travel distance parameter $\Psi$, as discussed in the previous section. The second, is implemented in GHPM by adding a progressive linear vaccination policy. More precisely, we pick random, not currently infected individuals, and transfer them to a permanent recovered state $R^*$. The vaccination policy is not started at $t=0$, but only after $t=365$ days in our 5-year long simulation framework (this approach mirrors the current COVID-19 situation better). Afterwards, a random set of individuals are selected every single iteration as the number of immune individuals grows progressively.

**Figure 5:**
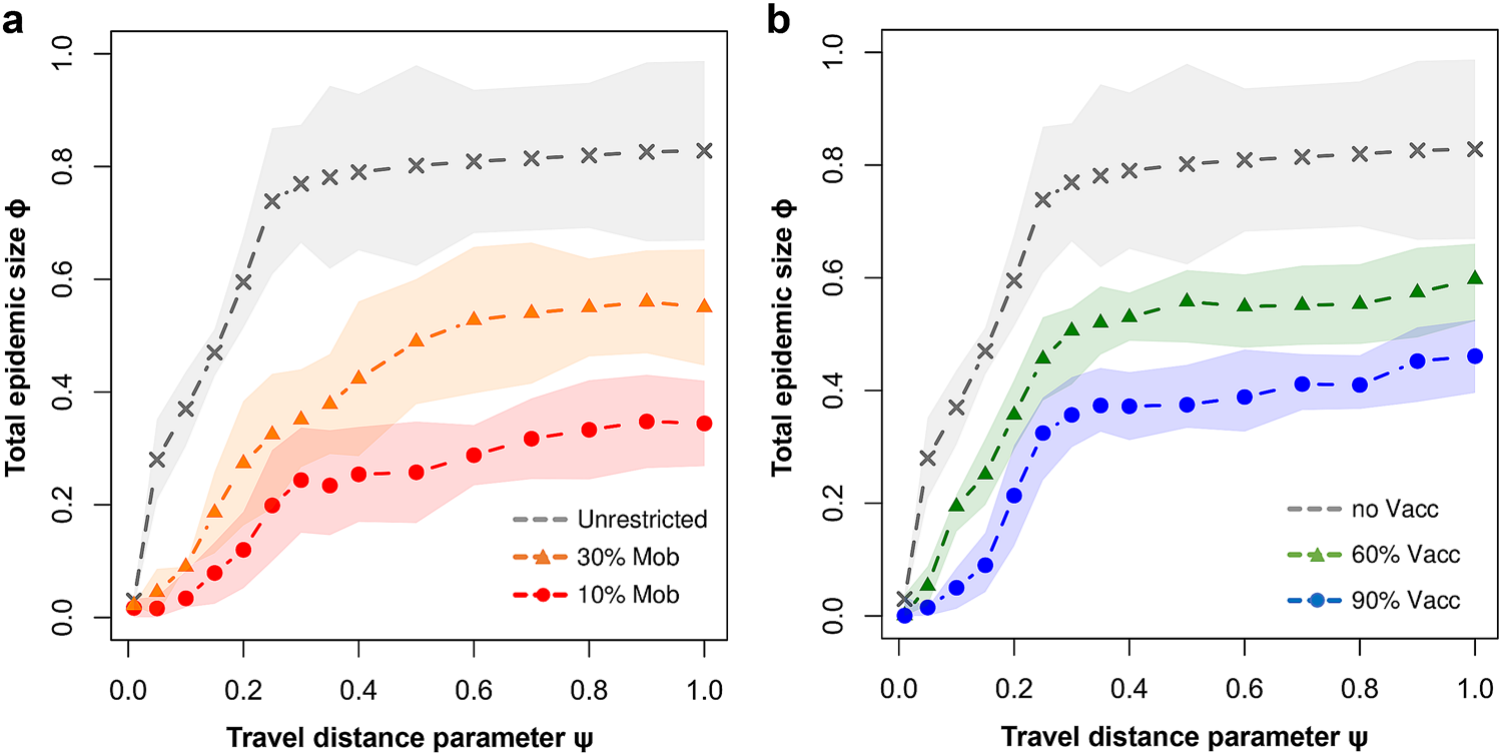
Total epidemic size $\phi$, averaged over 2000 simulations, as a function of the travel distance parameter $\Psi$ in two complementary scenarios. (**a**) Epidemic size in response to a mobility reduction down to 30% (orange), respectively 10% (red). (**b**) Epidemic size in response to adopting a linear immunization policy of vaccinating 60% (green), respectively 90% (blue) of individuals per year of simulation (i.e., 365 iterations). Colored regions indicate 95% CI.

Figure 5a depicts the impact of two mobility restriction policies of reducing travel to 30% (orange line), respectively to just 10% (red line) of the original, unrestricted travel. We note that, for increasing $\Psi >0.2$, even very strict measures do not guarantee a proportional reduction in epidemic size $\phi$.

Figure 5b summarizes the impact of two immunization policies of vaccinating individuals at a rate of 60% per year (5%/month; green line), respectively 90% per year (7.5%/month; red line), compared to no vaccination at all (gray line). Here we observe that, as the travel distance parameter $\Psi$ increases, even an aggressive vaccination policy is unable to guarantee a proportional reduction in epidemic size $\phi$. Of course, this is, in part, a consequence of our choice to implement the vaccination policy with a 1 year delay in our simulations. Further experimenting with different vaccination delays, or vaccination rates is outside the scope of this paper but can make an interesting follow-up research topic.

## Discussion

The introduction of our multiscale GHPM model enables us to reveal several meaningful characteristics of real-world epidemic outbreaks that are, otherwise, challenging to describe with homogeneous mixing models which adopt a single scale^15–19^. For instance, most epidemic spreading models describe any outbreak though only two outcomes: (1) an epidemic trigger condition is not fulfilled and the disease subsides in a local sub-population, or (2) the condition is fulfilled, and the disease manages to spread globally to a large scale comparable to the entire population^47^. The epidemic sizes distribution always becomes bimodal, as the first mode correlates to unsuccessful, local outbreaks, and the second mode correlates to successful, global scale outbreaks (see Fig. 3a,d). Different, network science approaches have also been used in computational epidemics, like a forest-fire topology^48^, and social networks with two^49^ and multiple dimensions^50^. However, even relatively complicated network models lead to the same bimodal distributions.

The motivation of this study is to bridge realistic, hierarchical population structuring, with individual mobility patterns and infectious dynamics with patient relapse into a reliable simulation framework, targeting the better prediction and control of epidemic dynamics. As such, we propose a very fine-grained population structuring and mobility influenced by spatial and hierarchical constraints. To the best of our knowledge, our approach is novel, and we provide qualitative comparisons to homogeneous population mixing through means of epidemic size distributions. Furthermore, our empirical data restates the important temporal heterogeneity of many large epidemics that has yet to receive full attention in the modeling state of the art. This heterogeneity is exemplified in our real data on influenza and COVID-19 evolution (Fig. 1), and reproduced by our GHPM model (Fig. 3).

A predominant body of the disease epidemiology state of the art focuses on $R_0$ as the central topic of research^8,12,23,51^. While this number has an inherent value for compartmental models with homogeneous mixing, $R_0$ could also be estimated for a more complex deterministic multiscale model. Nevertheless, estimating or redefining $R_0$, would not improve any relationship to the final epidemic size in stochastic multiscale models^20^, like our GHPM. The large, non-deterministic variations in epidemic size (Fig. 3e,f), and the resurgent characteristic (Fig. 3b,c) do not result from initial conditions like $R_0=\lambda /\mu$, but rather from “black swan”^52^ events, during which an infected individual travels from a compromised household (or settlement) to new uninfected settlements. In this way, the fluctuating yearly epidemic size $\phi _y$ is mostly decided by the increasing travel distance and travel frequency to susceptible populations.

In addition, we show that, apart from rare, long distance travel events, the population structure is a decisive factor in influencing the speed and impact of epidemic outbreaks. For example, the recent COVID-19 epidemic has a very varied evolution across countries (suggested by the distribution in Fig. 1b), ranging from less than 1–10% prevalence in 2020. These variations can also be attributed to the intensity, delay, and strength of implementing restrictions^23^. Even though non-pharmaceutical interventions (NPIs) are a relevant form of active control on epidemic duration and size^9^, our observations pinpoint that the GHPM population organization can achieve stochastic dynamics in epidemic size as large and heterogeneous as empirically observed fluctuations.

The focus of our simulation experiments was to determine how population organization, travel distance and travel frequency affect the spread of disease. In this sense, we show that travel restrictions, like reducing $\Psi$, and $\Theta$ (or equivalently $\Theta _h$) in our model (distance and frequency), can determine a significant change in the resulting epidemic size $\phi _y$, comparable to stronger social intervention strategies like vaccination or total quarantine. Specifically, we find a phase transition from local to global epidemic around $\Psi >0.1$ and $\Theta _h>1$. When the two parameters are close to 0, infected individuals are more likely to remain in their original household, and the resulting epidemic is delimited to the local scale ($h_{size}\ll \Omega$). Overall, we conclude that minor modifications in $\Psi$ have a higher impact on the size of the epidemic $\phi _y$ compared to similar modifications in $\Theta _h$.

One of the main take-away messages of this study is that we found travel distance to be more significant in increasing epidemic size than travel frequency. Additionally, our results pinpoint that, even an aggressive immunization policy (e.g., vaccination of 60-90% non-infected individuals per year), is unable to guarantee an immediate proportional reduction in epidemic size, given a delay of one year to start the vaccination campaign. To this end, vaccination (at a moderately realistic pace) does not yield lower epidemic sizes than reduced travel distances.

To conclude, major recent outbreaks like Ebola, SARS or COVID-19 repeatedly confront public health authorities with the uncertainty of—how big will it be this time? Unfortunately, current state of the art computational epidemiology can hardly offer accurate answers. Even the most complicated models of infectious spreading require an estimation of the relevant susceptible population. It is much easier to do retrospective studies in which, after an epidemic is observed, the specific parameters are approximated. However, such an approach is often limited because it has reduced relevance for future outbreaks. For instance, the global 1918 Spanish flu pandemic did not stop the 2003 SARS or 2020 COVID-19 pandemics. Even if the planet was less populated and less connected a century ago, the 1918 flu made considerably more victims compared to the potentially more infectious diseases of the XXI century. With the statistics of SARS pointing towards a quick mitigation with relatively low global impact, COVID-19 has had a very different evolution^53^. In the current pandemic context, the population susceptible to the SARS-CoV-2 virus is roughly the entire population of the planet. Should we then estimate scenarios with outbreaks concentrated around large populated hubs, or include billions of susceptible individuals spread across the entire planet? Perhaps the ultimate question is simply formulated as—what is the epidemic size distribution for a given infectious pathogen?

Our GHPM model tries to address these questions, to the best possible extent, by structuring population as a geographically spaced hierarchical set of sub-populations, modeled down to the level of households. The major advantage over other single scale, multiscale, or network models is that it can be extended to very large scales (e.g., continental or global) without implying homogeneous populations. This way, a modeled epidemic can be seen as multiple smaller epidemics occurring at different times, in different sub-populations. In this context, most of the infectious spreading happens locally, and global spreading is determined by rare distant travel. Because the epidemic size distribution is remarkably susceptible to the population structure, we suggest that epidemic control can be improved through adequate strategies applied to the boundaries inherently delimiting large, multiscale populations. We believe that future studies can adopt and extend our concept of geo-hierarchical population mobility to study progressively more realistic epidemic models of infectious spreading.

## Methods

### Geo-spatial population data

GHPM supports a pseudo-realistic organization of the population on which to run an epidemic outbreak simulation. In this sense, the settlements number |*S*|—and implicitly the total population of the experiment $\Omega$—are defined by a chosen geographical area *A*, most commonly limited to a country. In this sense, the number, position and size of settlements are defined according to data extracted from the Global Rural-Urban Mapping Project (GRUMP v1), revision 01 (March 2017) curated by the Center for International Earth Science Information Network (CIESIN), Columbia University^54^. GRUMP is an undergoing large-scale project, and is still missing some data, to a variable extend, for some countries. For the purpose of this paper we run all experiments on a model of Germany, with with 53.31M inhabitants (as defined by GRUMP) spread over 1132 settlements, which we consider a mid-sized representative example.

Inside each settlement, GHPM uses a simplified synthetic, but intuitive hierarchical organization of inhabitants using 2–5 levels of administrative units. We chose the synthetic alternative (inside settlements) because of the limitation in available data defining the organization of each settlement. Nevertheless, with more available data in the future, GHPM can be modified to offer a more precise mapping of each settlement’s population. Conversely, GHPM can be used without any real data, if one so chooses, by creating a synthetic set of settlements, defined by positions and populations according to any distribution of choice.

### GHPM experimental setup

In order to significantly accelerate the large number of GHPM experiments, we chose to scale down the modeled population by 100 fold. As such, the final simulation population used throughout the experiments is 533,160 individuals (i.e., 1% of uniformly scaled down population of Germany from the GRUMP dataset).

In general, the size of each unique household $h_i$ may be chosen as a uniformly distributed integer number between 1–4 individuals (i.e., average $h_{size}=2.5$) based on UN data for developed countries^42^. In particular, we use a custom distribution of household sizes according to data available in 2019 for Germany (Federal Statistical Office—Statistisches Bundesamt), as detailed in SI.1.

A branching factor $\beta$ can be used to determine the number of divisions in a settlement, based on the number of households. In this sense, a static parameter of $\beta =0.5-0.7$ makes a good approximation of the hierarchical density of administrative units. In this paper we use $\beta =0.6$. Overall, it makes practical sense to use $1>\beta >0.5$ (i.e., larger than square root) in order to obtain a more dense hierarchy of upper level divisions. A sensitivity analysis for $\beta$ is provided in SI.2.

All simulations run for $t=2000$ iterations (days). If we consider the correspondence 1 iteration = 1 day, then the simulation duration translates to $2000/365 \approx 5.48$ years. Since the target of our study are resurgent epidemics (with relapse), the final epidemic duration and final size cannot be expressed in absolute values (they run indefinitely in most cases). Our approach, throughout the paper, is to use the yearly epidemic size $\phi _y$ as a measure of the repeating outbreak waves. Intuitively, we average the total epidemic size $\phi$ over the simulation period, i.e., $\phi _y=\phi /5.48$. Furthermore, the impact of each parameter in the GHPM model is discussed in SI.3.

The timeout period for any recovered individual is uniformly chosen between of 3–6 months (current estimates of COVID-19 immunity of 3 months^55^, 4–5 months^56^, 6 months^57^). The adopted infection rate is $\lambda =0.06$, and the recovery rate is $\mu =0.02$. While a theoretical reproduction number $R_0$ is based on the $\mu$ and $\lambda$, in practice^22^, we estimate $R_0$ from the household size distribution, and obtain $R_0=0.983$. Recent studies suggest an $R_0=3.30 \pm 1.4$ for COVID-19^51^.

## Supplementary Information

Supplementary Information.

## Data Availability

COVID-19 data used in this study are supplied by the European Centre for Disease Prevention and Control (https://www.ecdc.europa.eu/en/covid-19/data). Weekly influenza data are supplied the US Outpatient Influenza-like Illness Surveillance Network (ILINet) (https://gis.cdc.gov/grasp/fluview/fluportaldashboard.html), by Google Trends (https://www.google.com/publicdata/explore?ds=z3bsqef7ki44ac_) . Measles cases in Indonesia are supplied by the International Federation of Red Cross and Red Crescent Societies (IFRC) (https://data.humdata.org/m/dataset/indonesia-measles-outbreaks-2015-2017?force_layout=light). H1N1 data are provided by the WHO via Kaggle (https://www.kaggle.com/de5d5fe61fcaa6ad7a66/pandemic-2009-h1n1-swine-flu-influenza-a-dataset). Pertussis data are provided by the California Department of Public Health (CDPH) via Health.gov (https://healthdata.gov/State/Vaccine-Preventable-Disease-Cases-by-County-and-Ye/58x3-zrxa).
