# Supplementary material for: A novel geo-hierarchical population mobility model for spatial spreading of resurgent epidemics
Source: Sci Rep. 2021 Jul 12;11:14341. doi: 10.1038/s41598-021-93810-8 (PMC8275763; doi:10.1038/s41598-021-93810-8)
Supplement: Supplementary file 1 — Supplementary Information. [file 41598_2021_93810_MOESM1_ESM.pdf]

Supplementary Information

A Novel Geo-Hierarchical Population Mobility Model for Spatial Spreading of Resurgent Epidemics

Alexandru Topîrceanu, and Radu Emil-Precup

SI.1. Real-world household size distribution

As a general advice, the size of each unique household h_i, initialized in our GHPM model, may be chosen as a uniformly distributed integer number between 1–4 individuals (i.e., average $h_{size} = 2.5$) based on UN data for developed countries[1].

Nevertheless, our particular focus on reproducing a reliable GHPM model for Germany determines us to define the distribution of household sizes according to data available in 2019 for Germany. Data were obtained from two sources: the Federal Statistical Office - Statistisches Bundesamt (Available at https://www.destatis.de/EN/Themes/Society-Environment/Population/_Graphic/_Interactive/households-families-size.html) and Statista ("Number of households in Germany from 2000 to 2019, by size (in 1,000)") (Available at: https://www.statista.com/statistics/464187/households-by-size-germany/). Table 1 provides the detailed distribution of households and their estimated number based on the estimated average household size and the number of households in the model. Specifically, the model of Germany has 533,160 inhabitants (as defined by GRUMP and reduced 100-fold), and $h_size = 1.98$ results from the probabilities in Table 1. Given h_size, we estimate a number of 533,160/1.98 = 269,720 households.

Table 1. Distribution of household sizes (1-5 individuals) used in the GHPM model for Germany. The statistical estimation of household numbers of each size is based on the total number of households of 269,720.

Household size	Probability	Number in model
1 individual	0.423	113,902
2 individuals	0.332	89,398
3 individuals	0.119	32,043
4 individuals	0.091	24,503
5 individuals	0.035	9,424

SI.2. Branching factor sensitivity analysis

We define the branching factor β to determine the number of divisions, for each level, throughout a settlement, based on the number of households. Specifically, starting from the estimated number of households in a settlement $n_h = \Omega^*(s_i)/h_{size}$, each upper-level administrative division is defined by raising the lower-level division at the power β.

We further present a detailed analysis of the impact of β from four perspectives:

- Fixed household number ($n_h = 100,000$) and variable branching factor ($\beta = 0.1 - 0.9$).
- Fixed branching factor ($\beta = 0.6$) and variable household number ($n_h = 30 - 300,000$).
- Fixed household number ($n_h = 100,000$) and decreasing branching factor across divisions ($\beta = 0.1 - 0.9$ which decreases by $\times 0.8$, for each level, upwards).
- Fixed household number ($n_h = 100,000$) and increasing branching factor across divisions ($\beta = 0.1 - 0.9$ which increases by $\times 1.2$, for each level, upwards).

Tables 2-5 summarize the results for the above described scenarios. In Tables 2-3 we observe an expected high sensitivity of the GHPM model's hierarchical density based on β. Namely, for an average sized city ($n_h = 100,000$ with $\approx 200,000$ inhabitants) we obtain more than two neighborhoods $n_n \geq 2$ only starting from $\beta \geq 0.5$. For high values of $\beta > 0.7$ the number of districts/neighborhoods becomes higher than expected in real-world settlements. Thus, a "sweet-spot" for the branching factor is around 0.5–0.7. Table 4 exemplifies the gradual reduction of β by a factor of $\times 0.8$ from blocks upwards to districts. This reduction clearly reduces the density of higher level administrative units; only a $\beta \geq 0.7$ is practical for settlements of at least $n_h = 100,000$. Finally, Table 5 exemplifies the gradual increase of β by a factor of $\times 1.2$ from blocks upwards to districts. Here, only a $beta \leq 0.5$ makes practical sense; conversely, we obtain more upper-level divisions than lower-level ones (see values in italics in Table 5). We find that gradually increasing or decreasing β suggests both an upper limit of $\beta \leq 0.5$ and a lower limit of $\beta \geq 0.7$ which makes us conclude that a dynamical branching factor is less realistic.

Table 2. Number of upper-level administrative divisions in an example settlement with a fixed household number ($n_h = 100,000$) and a variable branching factor ($\beta = 0.1 - 0.9$).

β	**Blocks** n_b	**Streets** n_s	**Neighborhoods** n_n	**Districts** n_d
0.1	3	1	1	1
0.2	10	2	1	1
0.3	32	3	1	1
0.4	100	6	2	1
0.5	316	18	4	2
0.6	1,000	63	12	4
0.7	3,162	282	52	16
0.8	10,000	1,585	363	112
0.9	31,623	11,220	4,416	1,908

Table 3. Number of upper-level administrative divisions in an example settlement with a fixed branching factor ($\beta = 0.6$) and a variable household number ($n_h = 30 - 300,000$).

β	**Blocks** n_b	**Streets** n_s	**Neighborhoods** n_n	**Districts** n_d
30	8	3	2	2
100	16	5	3	2
300	31	8	3	2
1,000	63	12	4	2
3,000	122	18	6	3
10,000	251	28	7	3
30,000	486	41	9	4
100,000	1,000	63	12	4
300,000	1,933	94	15	5
1,000,000	3,981	145	20	6

Table 4. Number of upper-level administrative divisions in an example settlement with a fixed household number ($n_h = 100,000$) and a decreasing branching factor across divisions ($\beta = 0.1 - 0.9$ which decreases by $\times 0.8$, for each level, upwards – from blocks to districts).

β	**Blocks** n_b	**Streets** n_s	**Neighborhoods** n_n	**Districts** n_d
0.1	3	1	1	1
0.2	10	1	1	1
0.3	32	2	1	1
0.4	100	4	1	1
0.5	316	10	2	1
0.6	1,000	28	4	2
0.7	3,162	91	8	2
0.8	10,000	363	20	3
0.9	31,623	1,738	73	7
β-*modifier*	$\times 1$	$\times 0.8$	$\times 0.8$	$\times 0.8$

Table 5. Number of upper-level administrative divisions in an example settlement with a fixed household number ($n_h = 100,000$) and an increasing branching factor across divisions ($\beta = 0.1 - 0.9$ which increases by $\times 1.2$, for each level, upwards – from blocks to districts). Values in *italics* represent impossible numbers because one cannot have more upper-level divisions than lower level ones.

β	**Blocks** n_b	**Streets** n_s	**Neighborhoods** n_n	**Districts** n_d
0.1	3	1	1	1
0.2	10	2	1	1
0.3	32	3	2	1
0.4	100	9	4	3
0.5	316	32	12	9
0.6	1,000	145	74	*87*
0.7	3,162	871	*919*	*3,841*
0.8	10,000	6,918	*26,525*	*1,303,951*
0.9	31,623	*72,444*	*1,988,672*	*6,244,899,087*
β-modifier	$\times 1$	$\times 1.2$	$\times 1.2$	$\times 1.2$

As a conclusion of our analysis, we adopt a static parameter of $\beta = 0.6$ for all levels of administrative divisions.

SI.3. Impact of model parameters on epidemic size

We analyze the relationship between the following pairs of GHPM model parameters:

- Travel distance parameter Ψ (Psi) and epidemic size ϕ (Phi),
- Travel frequency parameter Θ (Theta) and epidemic size ϕ (Phi),
- Average number of transiting individuals per household Θ_h and epidemic size ϕ (Phi),
- Branching factor β (beta) for divisions and epidemic size ϕ (Phi),
- Average household size h_{size} and epidemic size ϕ (Phi),
- Inter-settlement travel probability P_1 and the travel frequency parameter Θ (Theta).

Similar to the already described methodology, all simulations run for $t = 2000$ iterations, and we keep all parameters to their default value (except the one being subject to testing), i.e., the default parameters values are: $\Psi_d = 0.2, \Theta_d = 1, \beta_d = 0.6, h_{size-d} = 1.98$.

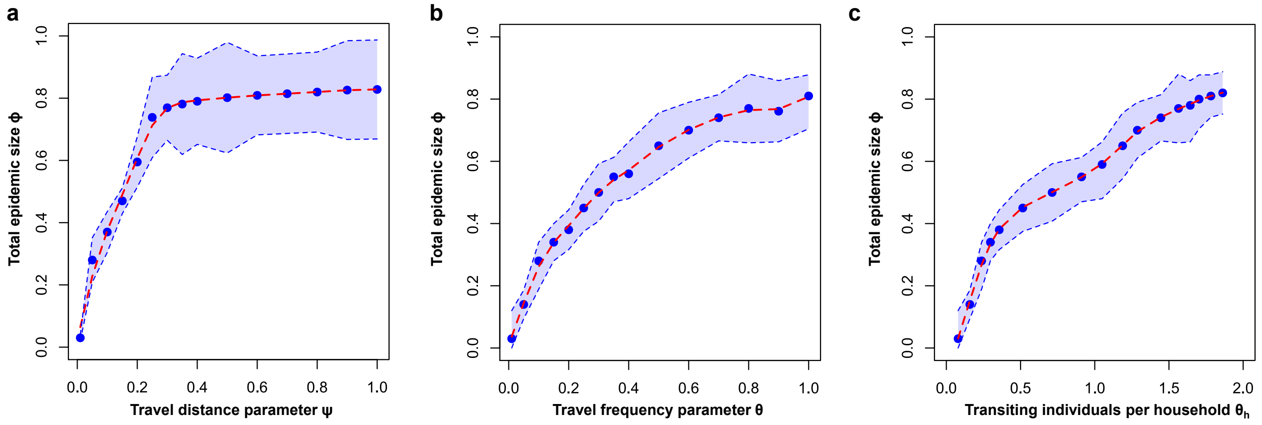

Figure 1. Total epidemic size ϕ as a function of (a) the travel distance parameter Ψ, (b) the travel frequency parameter Θ, and (c) the average number of transiting individuals per household Θ_h (on log scale). All blue regions indicate the 95% CI.

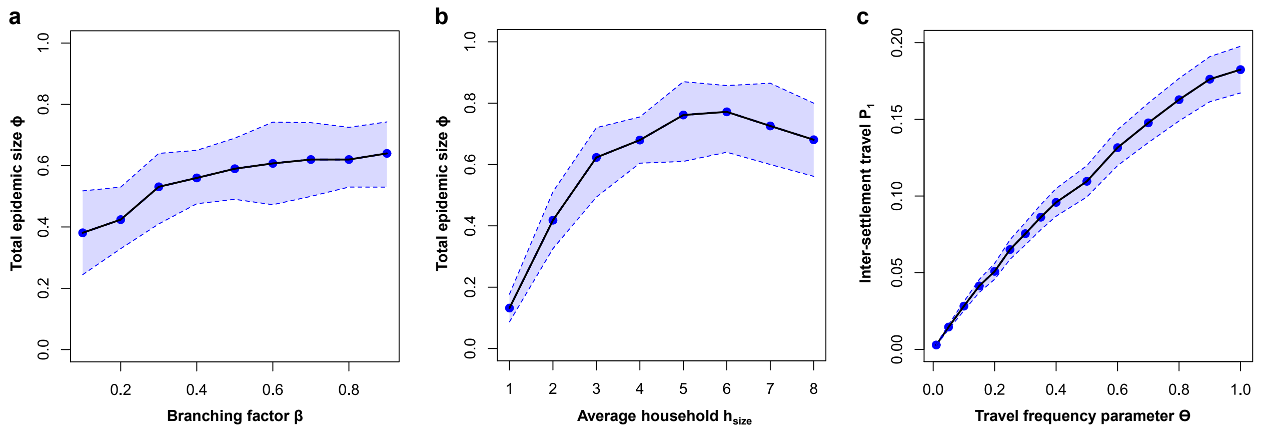

Figure 2. Total epidemic size ϕ as a function of (a) the administrative divisions branching factor β, (b) the average household size h_{size}, and (c) the inter-settlement travel probability P_1 as a function of the travel frequency Θ. All blue regions indicate the 95% CI.

The GHPM model exhibits a higher sensitivity of the epidemic size (i.e., the model's main output) towards the travel distance parameter Ψ than towards the travel frequency parameter Θ (see Figure 1a-b). Furthermore, when estimating (through simulation) the average number of transiting individuals per household $Theta_h$, we observe a similar phase transition (see Figure 1c). Nevertheless, the transition in epidemic size ϕ triggered by Ψ happens sooner, for smaller values of Ψ, compared to Θ_h. Note that in Figure 1c we use log-scale on the OX axis.

Figure 2a suggests that the model output has a positive linear correlation with the branching factor β. Figure 2b suggests that the average household size h_{size} has a significant impact on ϕ. We tested values of h_{size} up to extremes (1-8); recent surveys and census data pinpoint towards the largest average households in Sub-Saharan Africa (6.9), and North-Africa/Middle-East (6.2), with exceptional cases being Benin (8.9) and Gambia (13.8)[2,3]; the smallest average households are found in Northern Europe (< 2). Figure 2c confirms a linear-like relationship between the travel frequency parameter Θ and the empirically measured inter-settlement travel probability P_1.

References

1. Nations, U. Household size and composition around the world. *Econ. Soc. Aff.* (2017).

2. Bongaarts, J. Household size and composition in the developing world in the 1990s. *Popul. studies* **55**, 263–279 (2001).

3. ***. Religion and living arrangements around the world. pew research center analysis of 2010-2018 census and survey data. *Pew Res. Cent.* (2018).
